# Gated subspace alignment with drift compensation for parameter-efficient Class-Incremental Learning

**DOI:** 10.1371/journal.pone.0348270

**Published:** 2026-05-07

**Authors:** Jianye Gu, Shucheng Huang, Tian Li, Senbao Zhang, Mingxing Li

**Affiliations:** 1 School of Computer, Jiangsu University of Science and Technology, Zhenjiang, Jiangsu, China; 2 Suzhou Institute of Technology, Jiangsu University of Science and Technology, Suzhou, Jiangsu, China; 3 Jingjiang College, Jiangsu University, Zhenjiang, Jiangsu, China; Universidad CEU Cardenal Herrera - Campus Elche, SPAIN

## Abstract

Class-Incremental Learning (CIL) aims to enable models to continuously learn new categories while preserving existing knowledge and avoiding catastrophic forgetting. Although parameter-expansion architectures can alleviate task interference to some extent, the representations of previously learned classes often drift or degrade as the feature subspaces continuously evolve and expand, resulting in decreased recognition performance for old classes. To address this issue, we propose an efficient CIL method—Dynamic Gated Adapter for Subspace Alignment (DGASA). Based on a frozen pre-trained backbone, DGASA introduces lightweight adapters with attention-based gating for each task to construct task-specific subspaces, while dynamically fusing cross-task information via attention mechanisms. In addition, DGASA learns a linear mapping between the old and new subspaces to achieve consistent alignment of old class prototypes in the current subspace without accessing past data. Extensive experiments demonstrate that DGASA significantly improves classification accuracy and resistance to forgetting on multiple benchmark datasets, offering strong generalization and computational efficiency.

## Introduction

Class-Incremental Learning (CIL) [1], as a core task in the field of continual learning, aims to enable models to continuously acquire new categories while effectively preserving and leveraging previously learned knowledge. This capability is essential for building intelligent systems with long-term and stable learning abilities. However, directly fine-tuning neural networks on new data often causes the model to forget previously acquired information, leading to a significant drop in performance—a phenomenon known as catastrophic forgetting [2,3]. To alleviate this issue, some studies have proposed parameter-expansion-based dynamic architectures [4–9], which introduce independent parameter modules for different tasks, effectively reducing task interference. These approaches have achieved promising results, particularly when combined with powerful pre-trained models.

In non-pretrained model settings, some expandable networks [4,8,9] construct separate backbone networks for each task, thereby forming task-specific feature subspaces and achieving effective isolation between tasks. These methods are relatively effective at preserving knowledge from previous tasks. However, as the number of tasks increases, the model’s parameter size and computational overhead grow rapidly, significantly affecting inference efficiency. Moreover, they often rely on retaining samples from previous tasks to train a unified classifier, which poses substantial challenges in real-world applications where data privacy and storage are constrained.

In contrast, pretrained models, with their powerful representational capacity and strong transferability, offer a more promising solution for building low-cost continual learning systems that do not require access to previous samples [9–15]. To fully leverage the advantages of pretrained models, many approaches [16–18] draw inspiration from expandable networks by designing lightweight modules to adapt to new tasks and mapping the ever-growing feature space to classifiers corresponding to each category. This strategy balances the recognition of both old and new classes, alleviating inter-task conflicts to some extent and improving the model’s adaptability and scalability. However, due to the complex interference among task-specific features and the dynamic shifts in feature distributions during training, these models still struggle to fully prevent catastrophic forgetting.

**Fig 1:**
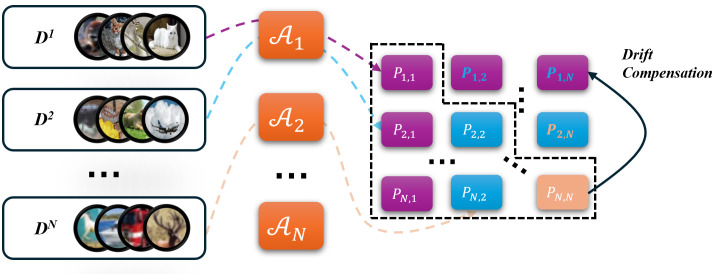
DGASA architecture: lightweight subspace construction and drift compensation.

To this end, we introduce DGASA (Dynamic Gated Adapter for Subspace Alignment), which achieves a balance between task isolation and knowledge sharing via lightweight subspace construction coupled with drift compensation, as shown in Fig 1. Specifically, DGASA freezes the pretrained backbone and inserts attention-gated adapters for each task, constructing low-dimensional subspaces and dynamically fusing them via an attention mechanism. This effectively alleviates inter-task conflicts and improves parameter efficiency. To tackle the issue of old class prototypes becoming invalid due to changes in the feature space during training, DGASA learns a linear mapping between new and old subspaces, enabling consistent reconstruction of old class prototypes in the current subspace without accessing old data, thereby maintaining stable representations. During inference, DGASA introduces an instance-level weighting strategy that dynamically fuses features based on the sample’s matching degree across subspaces, which strengthens features related to the current task while preserving the discriminative power of old knowledge. This significantly enhances the model’s generalization and incremental learning performance. The main contributions of this work are as follows:

- We propose the DGASA framework, which combines lightweight adapters with a gating mechanism to construct task-specific subspaces and enable dynamic fusion, significantly alleviating inter-task interference and improving parameter efficiency.
- We introduce a subspace drift compensation mechanism that learns a linear mapping between new and old subspaces, enabling data-free consistent reconstruction of old class prototypes, thereby enhancing model stability and data privacy.
- We conduct extensive experiments on commonly used continual learning datasets to demonstrate the effectiveness of the DGASA method. Comparative results show that our approach achieves the best performance.

## Related work

### Class-incremental learning

Class-incremental learning aims to enable a model to effectively retain knowledge of previously learned classes while continuously receiving information about new classes [11,19,20], thus avoiding catastrophic forgetting. Existing methods mainly fall into three categories: regularization methods, replay methods, and dynamic network methods. Among them, regularization methods [21–24] introduce additional constraints during training to limit the update magnitude of critical parameters, maintaining stable representations of old tasks. Replay methods preserve a portion of samples from old tasks [8,25,26] or use generative models to synthesize old-class data [27,28], and jointly train them with new data to help the model retain previously learned knowledge. Dynamic network methods expand the model structure [4, 5, 6, 7, 8, 29–31], for example, by adding new neurons, layers, or task-specific modules, effectively isolating knowledge between tasks and thereby improving the model’s ability to jointly adapt to and learn from both old and new tasks.

### Pre-trained model-based CIL

Class-incremental learning based on pre-trained models (PTMs) [11,31–33] has become a research hotspot in recent years. With the development of pre-training techniques, an increasing number of methods incorporate PTMs into class-incremental learning to enhance model performance and generalization ability. These methods typically keep the pre-trained weights frozen and achieve lightweight parameter updates through prompt tuning [15–18], encoding new task features into a prompt pool to effectively alleviate forgetting. In addition, some approaches adopt model fusion or model merging strategies [9,31,34–37] by saving and integrating models from multiple training stages to further improve the retention of old knowledge. Prototype-based classification methods leverage the powerful representations of PTMs combined with nearest class mean (NCM) classifiers [19,38–40] to achieve stable recognition of old classes.

## Method

### Problem definition

Class-Incremental Learning (CIL) is a learning scenario where a model continuously learns to classify new classes to build a unified classifier [1]. Given a sequence of *B* training datasets denoted as $\left\{D^{1},D^{2},\ldots ,D^{B}\right\}$, where the *b*-th dataset is $D^{b}=\{(x_{i},y_{i}){\}}_{i=1}^{n_{b}}$ containing $n_{b}$ instances. Each instance $x_{i}\in {\mathbb{R}}^{D}$ comes from class $y_{i}\in Y_{b}$. Here, $Y_{b}$ is the label space of task *b*, and for $b\neq b^{\prime }$, $Y_{b}\cap Y_{b^{\prime }}=\emptyset$, meaning the classes across different tasks do not overlap. We follow the exemplar-free setting in [16], where no samples from old classes are saved. Therefore, at the *b*-th incremental stage, we only have access to data from $D^{b}$ for training. In CIL, our goal is to build a unified classifier for all seen classes $Y_{b}=Y_{1}\cup \cdots \cup Y_{b}$ as data evolves. Specifically, we want to find a model $f(x):𝒳\to Y_{b}$ that minimizes the expected risk:


$$f^{*}=\arg\min_{f\in ℋ}{𝔼}_{(x,y)\sim D_{t}^{1}\cup \cdots \cup D_{t}^{b}}II(y\neq f(x)),$$ (1)

where ℋ is the hypothesis space, II(·) is the indicator function, and $D_{t}^{b}$ denotes the data distribution of task *b*. Following typical PTM-based CIL works [16–18], we assume a pretrained model is available for initializing *f*(*x*). We decouple the PTM into a feature embedding $\Phi (\cdot ):{\mathbb{R}}^{D}\to {\mathbb{R}}^{d}$ and a linear classifier $W\in {\mathbb{R}}^{d\times |Y_{b}|}$. The embedding function Φ(·) refers to the final [CLS] token in ViT, and the model output is expressed as $f(x)=W^{T}\Phi (x)$. For clarity, we decouple the classifier as $W=[w_{1},w_{2},\ldots ,w_{\left|Y_{b}\right|}]$, where $w_{j}$ is the classifier weight for the *j*-th class.

### Model overview

**Fig 2:**
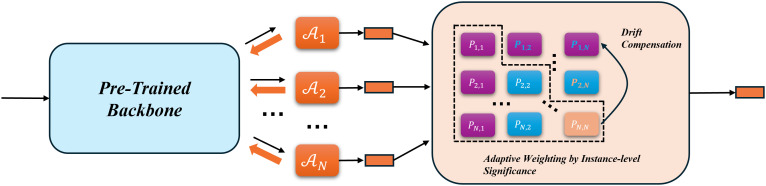
Inference pipeline of the proposed DGASA framework. GA modules are inserted in each Transformer block for task-specific adaptation.

Dynamic Gated Adapter for Subspace Alignment (DGASA) is an efficient framework specifically designed for class-incremental learning (CIL), with its overall workflow illustrated in Fig 2. In the base class training stage, DGASA freezes the pretrained backbone network, which serves as a shared feature extractor across all tasks. On top of this backbone, a set of Gated Adapter (GA) modules is introduced for each incremental task to construct task-specific embedding subspaces. This design enables effective task isolation and alleviates catastrophic forgetting.

As tasks incrementally progress, the feature subspaces continuously evolve and expand, potentially rendering old class prototypes ineffective in the current subspace. To address this, DGASA proposes a subspace drift compensation mechanism, which leverages prototype pairs of new classes generated from both the old and current subspaces as supervision signals. A linear mapping is then learned to explicitly project old class prototypes from their original subspace into the current one. This process requires no access to data from previous tasks, thereby maintaining prototype consistency while ensuring privacy friendliness.

During the inference stage, DGASA incorporates an instance-aware adaptive weighting mechanism to enable collaborative decision-making across multiple subspaces. Specifically, the model adjusts the contribution of each subspace to the final classification result based on how well the test sample matches the class prototypes in each subspace. The primary task subspace provides the main discriminative power, while the remaining subspaces are weighted based on their semantic saliency scores, leading to more refined and robust ensemble predictions.

In summary, DGASA integrates pretrained knowledge sharing, task-adaptive subspace modeling via GA modules, prototype mapping compensation, and saliency-aware inference into a unified multi-subspace incremental learning framework. Without requiring access to old data, it achieves excellent generalization, memory efficiency, and resistance to forgetting.

### Gated Adapter (GA) Module

In Class-Incremental Learning (CIL), a typical challenge lies in fine-tuning pre-trained models on new tasks without incurring significant computational overhead or suffering from catastrophic forgetting. Full fine-tuning of the entire model requires extensive computation and may cause the model to forget previously learned tasks. To address this, we propose a more parameter-efficient solution using adapter modules, which offer a lightweight way to incorporate task-specific knowledge into pre-trained models. Adapters are small bottleneck structures inserted within each Transformer layer, allowing task-specific adaptations while keeping the core model frozen.

Our approach builds on this concept and introduces a Gated Adapter (GA) module, which enhances the standard adapter’s flexibility by incorporating a gating mechanism to control activation dynamically. We clarify the naming: while the term attention-gated was used in earlier versions, the actual mechanism is a pooled linear gating rather than standard QKV attention. Specifically, each Transformer layer in the backbone network contains a feed-forward module with an additional gated residual branch, which is activated conditionally based on the input features. For an input feature $x\in {\mathbb{R}}^{d}$, the output is formulated as:


$$x_{o}=MLP(x)+g(x)\cdot \left(W_{\text{up}}\cdot \sigma \left(W_{\text{down}}\cdot x\right)\right),$$ (2)

where $W_{\text{down}}\in {\mathbb{R}}^{d\times r}$ and $W_{\text{up}}\in {\mathbb{R}}^{r\times d}$ are weight matrices that reduce and then expand the dimensionality, σ is a nonlinear activation function (GELU), and *g*(*x*) is the gating function defined as:


$$g(x)=\sigma (W_{g}\cdot Pool(x)),$$ (3)

where $W_{g}\in {\mathbb{R}}^{1\times d}$ is a linear transformation parameter, and Pool(x) performs global average pooling across the input feature dimensions. This pooling step captures high-level characteristics of the input, producing a scalar gate that adaptively weights the importance of the residual branch.

**Fig 3:**
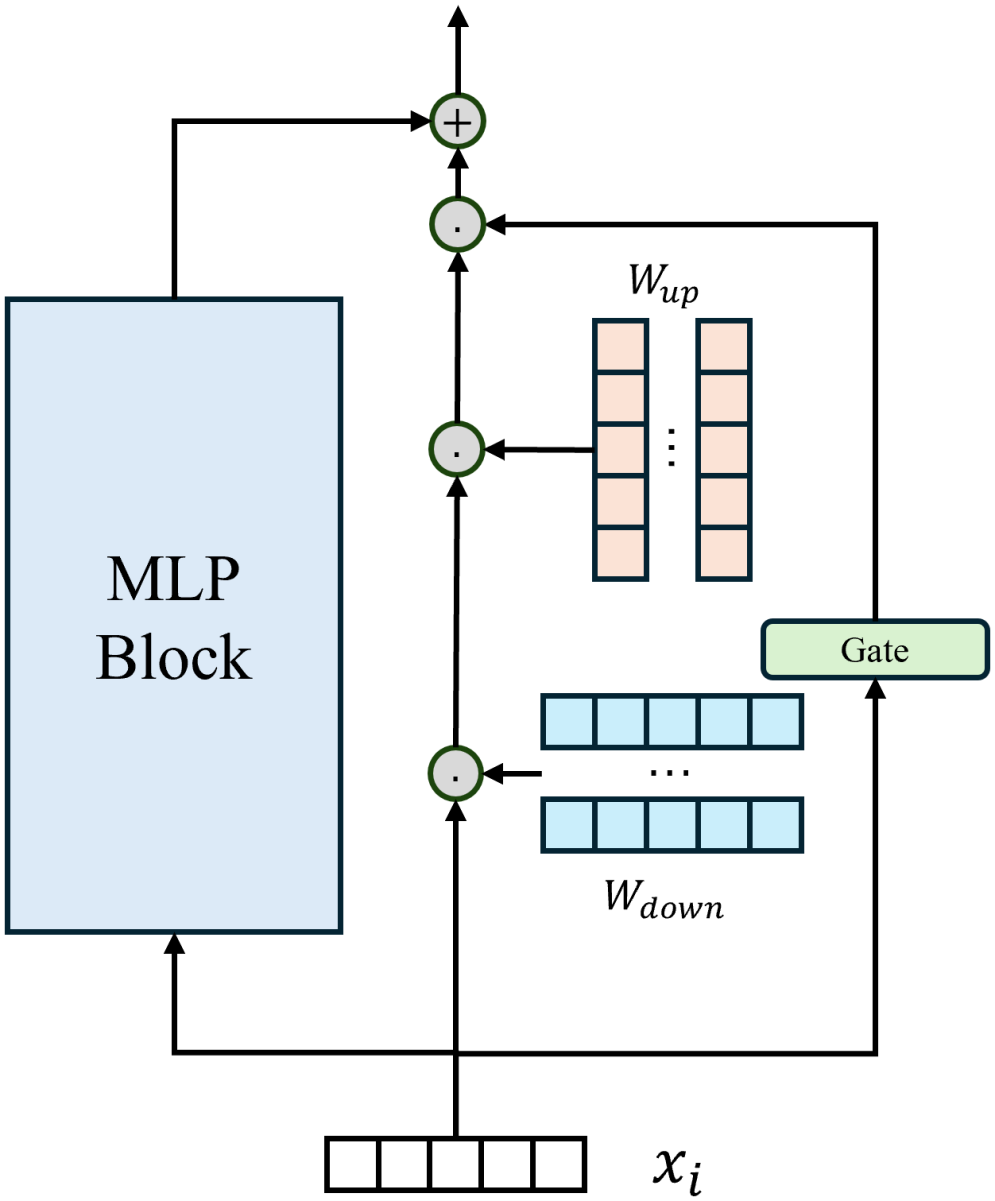
Architecture of the Gated Adapter (GA) module. The gating mechanism uses global pooling followed by a linear layer with sigmoid activation to produce a scalar gate *g*(*x*), which modulates the adapter residual branch.

Module vs. Framework Distinction. To avoid confusion, we explicitly distinguish:

- **GA module:** The individual gated adapter unit inserted in each Transformer layer (Fig 3). This is the basic building block for task-specific adaptation.
- **DGASA framework:** The complete method comprising: (1) a frozen pre-trained backbone, (2) a set of GA modules for each task, (3) the drift compensation mechanism for prototype alignment, and (4) the adaptive weighting mechanism for inference.

When the *b*-th task arrives, we introduce a new set of GA modules (one per Transformer layer) denoted as ${𝒜}_{b}$. As tasks progress, the model accumulates an adapter sequence $\left\{{𝒜}_{1},{𝒜}_{2},\ldots ,{𝒜}_{b}\right\}$. During inference, we concatenate the outputs of each task adapter to construct a joint feature representation:


$$\Phi (x)=[\phi (x;{𝒜}_{1}),\ldots ,\phi (x;{𝒜}_{b})]\in {\mathbb{R}}^{bd},$$ (4)

where $\phi (x;{𝒜}_{b})$ denotes the feature mapping through the GA modules of task *b*.

Since training for each task only optimizes its corresponding GA modules, learning new tasks does not affect knowledge retention of old tasks. Each GA module contains (2 *dr* + *d*) parameters, so the total storage cost is B×L×(2dr+d), where *B* is the number of tasks and *L* is the number of Transformer blocks (12 for ViT-B/16). With *d* = 768 and *r* = 16, each GA module has 25,344 parameters, totaling 304,128 parameters per task.

For classification prediction, we adopt a prototype-based classifier. After training the *b*-th task, we extract the prototype of the *i*-th class in the adapter subspace ${𝒜}_{b}$:


$$p_{i,b}=\frac{1}{N}\sum_{j=1}^{\left|D_{i}^{b}\right|}\phi (x_{j};{𝒜}_{b}),$$ (5)

where $D_{i}^{b}$ denotes the training sample set of class *i* in task *b*, and $N=|D_{i}^{b}|$. Then, the prototype vectors of this class in all task subspaces are concatenated to form the joint prototype:


$${𝒫}_{i}=[p_{i,1},p_{i,2},\ldots ,p_{i,b}]\in {\mathbb{R}}^{bd}.$$ (6)

During prediction, cosine similarity is used to compare the input feature embedding Φ(x) with the class prototypes ${𝒫}_{i}$, and the class with the highest similarity is chosen as the predicted label.

### Reconstructing prototypes under distribution drift

In incremental learning, as new tasks and distributions arrive, the model adapts by adding new adapters that construct embedding subspaces for each task. However, a challenge arises when it becomes necessary to recompute the prototypes for each class to ensure consistency with the current feature embedding space. Since accessing past data is often not feasible, directly computing the prototypes of old classes in the new subspace is problematic, leading to a mismatch between the prototype matrices at different stages of learning. This mismatch impacts the classifier’s ability to provide a unified and accurate representation of the feature space across all tasks.

To address this issue, we formalize it as a prototype completion task. Given two embedding subspaces (old and new) and two class sets (old and new), we aim to estimate the prototypes of old classes in the new subspace, denoted as $P_{o,n}\in {\mathbb{R}}^{|Y_{o}|\times d}$, by leveraging the following three observable prototype matrices: prototypes of old classes in the old subspace $P_{o,o}\in {\mathbb{R}}^{|Y_{o}|\times d}$, prototypes of new classes in the old subspace $P_{n,o}\in {\mathbb{R}}^{|Y_{n}|\times d}$, and prototypes of new classes in the new subspace $P_{n,n}\in {\mathbb{R}}^{|Y_{n}|\times d}$.

To reconstruct the prototypes, we adopt the concept of drift compensation [41], which models the geometric transformation between embedding subspaces. Unlike semantic-based approaches, this formulation does not rely on semantic similarity between classes, making it applicable even when new classes are sparse or semantically distant.

Specifically, we construct a paired sample set using the prototypes of new classes in both the old and new subspaces. These paired prototypes serve as anchors to estimate a linear mapping from the old subspace to the new subspace. We formulate the mapping estimation as a least-squares problem:


$$\min_{W}\|P_{n,o}W-P_{n,n}\overset{2}{\underset{F}{\|}},$$ (7)

which admits a closed-form solution via the normal equations:


$$W=(P_{n,o}^{T}P_{n,o}{)}^{-1}P_{n,o}^{T}P_{n,n}.$$ (8)

After obtaining the mapping matrix *W*, we reconstruct the prototypes of old classes by projecting the old subspace prototypes $P_{o,o}$ into the new subspace:


$${\hat{P}}_{o,n}=P_{o,o}W.$$ (9)

This projection does not require access to past data and enables consistent prototype alignment across tasks. In addition to downstream classification accuracy, we also evaluate the quality of drift compensation using direct metrics such as prototype reconstruction error and cosine similarity before and after alignment.

**Mapping Strategy:** It is crucial to clarify that the mapping matrix *W* is estimated **globally** with respect to all previously learned tasks, rather than being composed sequentially between adjacent tasks. Specifically, when a new task *b* arrives, the old subspace is defined by the concatenated feature space of all previous adapters $\left\{{𝒜}_{1},\ldots ,{𝒜}_{b-1}\right\}$, and the new subspace is defined by the concatenated space including the new adapter ${𝒜}_{b}$. The prototypes of the new classes ($P_{n,o}$ and $P_{n,n}$) are computed in these two respective subspaces and used to solve for a single, unified mapping *W*. This global mapping is then applied to all old class prototypes ($P_{o,o}$) to project them into the new subspace. This approach ensures that the mapping is solved only once per incremental session and avoids the potential for error accumulation that could arise from composing multiple sequential transformations. Computationally, solving this mapping requires $O(|Y_{n}|\cdot d^{2})$ operations per task, which is negligible compared to the cost of training the adapters.

**Numerical Stability and Robust Formulation.** In practice, the matrix $P_{n,o}^{T}P_{n,o}$ may be ill-conditioned or even singular, particularly when the number of new classes $\left|Y_{n}\right|$ is small relative to the embedding dimension *d*, or when the prototype matrix $P_{n,o}$ is rank-deficient. In such cases, directly computing the inverse may lead to numerical instability and degraded reconstruction quality.

To address this issue, we adopt a regularized least-squares formulation based on ridge (Tikhonov) regularization:


$$W_{\lambda }=(P_{n,o}^{T}P_{n,o}+\lambda I{)}^{-1}P_{n,o}^{T}P_{n,n},$$ (10)

where λ>0 is a regularization parameter and *I* is the identity matrix. This formulation improves numerical stability by ensuring that the matrix to be inverted is well-conditioned. In addition, we consider an alternative formulation based on the Moore–Penrose pseudoinverse:


$$W=P_{n,o}^{†}P_{n,n},$$ (11)

which provides a minimum-norm solution even when $P_{n,o}$ is rank-deficient.

In our implementation, λ is selected as a small constant (e.g., $10^{-4}$) and further validated via a sensitivity analysis. We empirically evaluate the robustness of the mapping with respect to λ, the number of new classes, and the conditioning of $P_{n,o}$.

After obtaining the mapping matrix *W*, we reconstruct the prototypes of old classes by projecting the old subspace prototypes $P_{o,o}$ into the new subspace:


$${\hat{P}}_{o,n}=P_{o,o}W.$$ (12)

This projection does not require access to past data and enables consistent prototype alignment across tasks. In addition to downstream classification accuracy, we also evaluate the quality of drift compensation using direct metrics such as prototype reconstruction error and cosine similarity before and after alignment.

### Adaptive weighting by instance-level significance

So far, we have introduced subspace expansion and adapter incremental learning mechanisms, and restored the prototypes of old classes through a prototype completion strategy. After completing adapter expansion and prototype completion, we construct a complete classifier with the following prototype matrix:


$$\left[\begin{array}{c} P_{1,1}{\hat{P}}_{1,2}\cdots {\hat{P}}_{1,B}\\ P_{2,1}P_{2,2}\cdots {\hat{P}}_{2,B}\\ \vdots \vdots \ddots \vdots \\ P_{B,1}P_{B,2}\cdots P_{B,B} \end{array}\right]$$ (13)

where the off-diagonal terms ${\hat{P}}_{b,i}$ above the main diagonal are completed according to the estimation formula.

During inference, to obtain the classification logits for the *b*-th task, we perform multiple prototype-embedding matches across different subspaces and aggregate them into the final score:


$${\left[P_{b,1},P_{b,2},\ldots ,P_{b,B}\right]}^{T}\Phi (x)=\sum_{i}P_{b,i}^{T}\phi (x;A_{i}),$$ (14)

where $\phi (x;A_{i})$ denotes the features extracted by the *i*-th adapter $A_{i}$. Although this ensemble approach helps leverage information from multiple subspaces, we note that only the adapter $A_{b}$ corresponding to the *b*-th task is specifically trained for that task, so the features $\phi (x;A_{b})$ are more task-discriminative.

To address this, we propose a new inference mechanism — Adaptive Weighting by Instance-level Significance. Its core idea is to quantify the matching degree of the input sample across different subspaces and dynamically adjust each subspace’s contribution weight to the final classification result based on this significance. Compared to using fixed scaling coefficients for all non-primary subspaces, our method is more flexible and better reflects the semantic correlation between the sample and each subspace.

The specific steps are as follows:

First, compute subspace significance. For a given input sample *x*, we input it into all adapters $A_{i}$ corresponding to the subspaces and extract feature representations $\phi (x;A_{i})$ for each subspace. Then, we calculate the similarity between these features and the corresponding prototypes $P_{b,i}$ to measure the matching degree of the sample in the *i*-th subspace. This matching degree is called the significance score $s_{i}(x)$, calculated as:


$$s_{i}(x)=sim(P_{b,i},\phi (x;A_{i})),$$ (15)

where sim(·,·) denotes a similarity function; here, we use cosine similarity. Note that to emphasize the primary subspace for the current task, we compute significance only for non-primary subspaces i≠b.

Next, normalize the significance scores into a weight distribution. To ensure comparability of contributions across subspaces, we apply the softmax function to the significance scores to obtain the weight distribution $\omega_{i}(x)$ for each non-primary subspace:


$$\omega_{i}(x)=\frac{\exp(s_{i}(x))}{\sum_{j\neq b}\exp(s_{j}(x))},\;i\neq b.$$ (16)

This normalization can be seen as an adaptive attention allocation over all non-primary subspaces, where higher significance scores correspond to larger weights, thereby enhancing the role of that subspace in the final inference.

Finally, construct the weighted classification score. After obtaining the primary subspace matching score $P_{b,b}^{T}\phi (x;A_{b})$, we combine it with the significance-weighted scores of all non-primary subspaces to obtain the final classification logits:


$$P_{b,b}^{T}\phi (x;A_{b})+\alpha \sum_{i\neq b}\omega_{i}(x)P_{b,i}^{T}\phi (x;A_{i}),$$ (17)

where α∈(0,1) is a balancing parameter controlling the relative contribution of non-primary subspaces in the overall score. In our experiments, we set α=0.15.

### Experiments

To analyze the impact of each component on the overall performance of the model, we performed ablation experiments on the Cifar-100 dataset for the Inc5 task, validating the effectiveness of the four key components proposed: PTM, Attention-Gated Adapter, Reconstructing Prototypes under Distribution Drift, and Adaptive Weighting by Instance-level Significance. The experimental results confirm the effectiveness of these methods. In this section, we first provide an overview of the datasets and evaluation metrics used, followed by a comprehensive description of the model architecture and experimental setup. We then present and analyze the results of the comparative experiments. Finally, we conduct ablation studies to validate the effectiveness of each component of the model.

## Datasets and evaluation metrics

### Datasets

Since pre-trained models may possess extensive knowledge from upstream tasks, they are often evaluated on a variety of datasets to assess their transferability and generalization capabilities in different learning scenarios. In this work, we follow the experimental setups in [18,19] to evaluate performance on several widely-used benchmark datasets, including CIFAR-100 [42], CUB-200 [43], ImageNet-R [44], ObjectNet [45], and OmniBenchmark [46]. These datasets are particularly useful for evaluating class-incremental learning (CIL) approaches, as they consist of a variety of task distributions and domain shifts. The datasets we use for evaluation include both standard CIL benchmarks and out-of-distribution datasets, offering a wide range of challenges that test the model’s ability to generalize across domains. CIFAR-100 consists of 100 classes, which are commonly used in incremental learning tasks. CUB-200, containing 200 classes, is widely used for fine-grained recognition and provides a higher level of class granularity. ImageNet-R and ObjectNet, each containing 200 classes, represent more challenging benchmarks, as they come from distribution shifts or domain gaps compared to the ImageNet dataset, which is typically used for pre-training. OmniBenchmark, with 300 classes, is another large-scale benchmark that provides diverse challenges in out-of-distribution testing, offering a more complex scenario for evaluating domain adaptation and generalization. These datasets provide a comprehensive testing ground for evaluating both generalization to new tasks and robustness in the face of domain drift.

**Dataset split:** To ensure consistency and comparability across different methods, we follow the established benchmark settings outlined in [1,18]. Specifically, we use the notation Inc-n to represent the class split, where *n* indicates the number of classes introduced at each incremental stage. In this framework, the first training stage starts with zero classes, meaning the model must progressively learn to recognize new classes as they are introduced. This incremental learning setup closely mirrors real-world scenarios where a model must continually adapt to new information without the luxury of accessing previous data. For a fair comparison across all methods, we adopt the same random seed for shuffling the class order before performing data splitting, as recommended in [1]. This ensures that the class order is randomized in a consistent manner across all experiments, preventing any potential bias introduced by a particular class ordering. Furthermore, we maintain consistency in the training and testing sets by aligning them with the settings used in [19] across all compared methods. This consistent dataset partitioning ensures a rigorous and fair evaluation of the methods under consideration, allowing us to draw reliable conclusions about their performance in incremental and out-of-distribution learning tasks.

### Evaluation metrics

Following the benchmark protocol [42], we use ${𝒜}_{b}$ to represent the model’s accuracy after the *b*-th stage. Specifically, we adopt ${𝒜}_{B}$ (the performance after the last stage) and


$$\bar{𝒜}=\frac{1}{B}\sum_{b=1}^{B}{𝒜}_{b}$$ (18)

(the average performance over all incremental stages) as evaluation metrics

### Implementation details

All experiments are conducted on a single NVIDIA RTX 4090 GPU (24 GB VRAM) using the PyTorch framework (version 2.0.1) [47]. Following prior works [18,19], we adopt a ViT-B/16 backbone pre-trained on ImageNet-21K (“vit_base_patch16_224” from the timm library, 86.6M parameters), and further fine-tuned on ImageNet-1K under the standard protocol. For all incremental tasks, DGASA is trained using the AdamW optimizer with $\beta_{1}=0.9$, $\beta_{2}=0.999$, and weight decay of 0.01. The initial learning rate is set to $5\times 10^{-4}$ and decayed by a factor of 0.1 at 50% and 75% of the total training steps. We use a batch size of 64 and train for 15 epochs per task with an input resolution of 224×224. The training objective consists of a cosine-similarity-based cross-entropy loss over the current task classes, with temperature τ=0.07, where class prototypes are computed as the mean feature representations of all samples belonging to each class in the current subspace. It employs global average pooling for gating, GELU activation in the adapter, sigmoid activation for the gate, and a dropout rate of 0.1 applied to the adapter output. For prototype-based alignment, prototypes are updated after each task, and the drift compensation mapping is solved using ridge regularization with $\lambda =10^{-3}$. The adaptive weighting coefficient is set to α=0.15, selected via validation on a held-out subset. To ensure robustness, all experiments are repeated with five random seeds (42, 1234, 5678, 91011, 121314), and we report the mean and standard deviation. The class order is consistently shuffled across all methods for each seed to ensure fair comparison.

### Comparisons with the State-of-the-arts

In this section, we provide a comprehensive evaluation of DGASA by comparing its performance with several state-of-the-art methods on five widely used benchmark datasets. The results, summarized in Table 1, show that DGASA achieves the best performance across all datasets, significantly surpassing existing methods, including CODA-Prompt and ADAM, when using the ViT-B/16-IN21K pre-trained model. Specifically, DGASA excels in both average accuracy across incremental stages $\bar{𝒜}$ and accuracy after the final incremental stage ${𝒜}_{B}$, outperforming the other methods by substantial margins.

**Table 1:** Comparison of different methods on five benchmark datasets. $\bar{𝒜}$ denotes average accuracy over incremental stages, and ${𝒜}_{B}$ is the accuracy after the last stage. The best performance is shown in bold. All methods are implemented without using exemplars.

Method	CIFAR Inc5		CUB Inc10		IN-R Inc5		ObjNet Inc10		Omnibench Inc30	
$\bar{𝒜}$	${𝒜}_{B}$	$\bar{𝒜}$	${𝒜}_{B}$	$\bar{𝒜}$	${𝒜}_{B}$	$\bar{𝒜}$	${𝒜}_{B}$	$\bar{𝒜}$	${𝒜}_{B}$
Finetune	37.60	19.71	25.88	13.25	21.41	10.65	18.69	8.56	23.55	10.37
Finetune Adapter	60.32	49.08	65.96	52.23	47.24	39.95	49.36	35.17	61.79	49.83
LwF	46.35	41.21	49.03	32.21	40.18	26.65	33.21	20.79	47.32	34.08
SDC	68.11	62.94	70.56	66.25	52.06	49.08	38.87	28.82	60.78	50.11
L2P	85.91	79.89	66.94	56.12	66.61	59.36	63.80	52.35	73.32	64.61
DualPrompt	87.91	81.33	77.52	66.61	63.39	55.32	59.39	49.40	74.01	65.63
CODA-Prompt	89.03	81.86	83.89	73.10	64.29	54.93	65.81	53.12	76.91	67.92
SimpleCIL	87.31	82.09	82.06	86.57	62.34	54.33	65.19	53.36	79.18	72.94
ADAM+Finetune	87.51	81.16	91.74	86.30	70.42	62.31	61.33	48.26	72.19	64.91
ADAM+VPT-S	90.26	84.31	91.80	86.36	66.47	59.12	64.36	52.34	79.39	73.50
ADAM+VPT-D	88.31	82.01	90.89	84.90	68.65	60.36	67.67	54.51	80.91	74.36
ADAM+SSF	87.52	81.73	91.55	85.92	68.77	60.33	68.89	56.37	80.26	73.75
ADAM+Adapter	90.31	84.95	91.96	86.48	72.06	64.09	66.91	55.02	80.42	74.11
EASE	91.49	85.77	92.19	86.77	78.25	70.50	70.77	57.80	81.05	74.76
**DGASA**	**92.09** ± **0.2**	**86.25** ± **0.3**	**92.36** ± **0.3**	**86.90** ± **0.3**	**79.62** ± **0.4**	**71.03** ± **0.4**	**71.71** ± **0.4**	**58.64** ± **0.5**	**81.90** ± **0.4**	**75.37** ± **0.4**

To ensure a fair comparison, we selected several representative class-incremental learning (CIL) methods based on pre-trained transformers (PTMs), including both recent and classical approaches. These methods include L2P [18], DualPrompt [17], CODA-Prompt [16], SimpleCIL [19], EASE [31], and ADAM [19]. In addition, we also compare DGASA to traditional CIL methods that are equipped with the same pre-trained models, such as LwF [23], SDC [41], iCaRL [1], DER [29], FOSTER [8], and MEMO [11]. These comparisons provide a comprehensive evaluation of DGASA’s performance against the most prominent methods in the field.

The results from Table 1 clearly demonstrate the superiority of DGASA across all five benchmark datasets: CIFAR Inc5, CUB Inc10, IN-R Inc5, ObjNet Inc10, and Omnibench Inc30. For instance, DGASA achieves the highest average accuracy $\bar{𝒜}$ and final stage accuracy ${𝒜}_{B}$ on CIFAR Inc5, with values of 92.11 and 86.25, respectively, outperforming the second-best method, EASE, by a notable margin. Similar trends are observed in the other datasets, where DGASA consistently outperforms the competing methods in both metrics. This highlights DGASA’s strong generalization ability and robustness across different incremental learning scenarios. In addition to comparing DGASA with other PTM-based CIL methods, we also evaluate its performance against traditional exemplar-based methods, as shown in Table 2. These traditional methods typically rely on storing a fixed number of exemplars for each class to mitigate catastrophic forgetting and maintain previous knowledge. For the comparison, we follow the standard practice in class-incremental learning by setting the number of exemplars to 20 per class, as in the method proposed by Rebuffi et al. [1]. Despite not utilizing exemplars, DGASA maintains competitive performance when compared to these exemplar-based methods. This is a significant achievement, as it shows that DGASA’s approach—which does not rely on memory replay or exemplar storage—can still effectively preserve knowledge and achieve superior performance across incremental learning tasks.

**Table 2:** Comparison of different methods with exemplar usage and accuracies on benchmark datasets.

Method	Exemplars	ImageNet-R	B0 Inc20	CIFAR B0 Inc10
iCaRL	20/class	72.31	60.58	82.31
DER	20/class	80.40	74.24	85.91
FOSTER	20/class	81.29	74.38	89.72
MEMO	20/class	74.50	66.59	84.01
**DGASA**	**0**	**82.54**	**77.33**	**92.51**

Overall, these results validate the effectiveness and efficiency of DGASA as a state-of-the-art method for class-incremental learning, demonstrating its ability to outperform both recent PTM-based approaches and traditional exemplar-based methods, all while avoiding the need for storing exemplars.

### Ablation study

To analyze the impact of each component on the overall performance of the model, we performed ablation experiments on the Cifar-100 dataset for the Inc5 task, validating the effectiveness of the four key components proposed: PTM, Gated Adapter (GA) module, Drift Compensation, and Adaptive Weighting by Instance-level Significance. The experimental results, as shown in Table 3, confirm the effectiveness of these methods. Among them, PTM refers to using a frozen pre-trained model solely as a feature extractor; GA indicates the introduction of Gated Adapter modules for task-specific subspace modeling; Drift Compensation refers to compensating for the drift of old class prototypes through the Reconstructing Prototypes under Distribution Drift mechanism; Adaptive Weighting dynamically adjusts the contribution of different subspaces during inference using the Adaptive Weighting by Instance-level Significance method.

**Table 3:** Ablation Study Results on CIFAR-100 Inc5 Task.

PTM	Adapter	Drift Compensation	Adaptive Weighting	CIFAR	B0 Inc5
✓	✗	✗	✗	70.32	60.45
✓	✓	✗	✗	88.56	78.73
✓	✓	✓	✗	90.41	82.15
✓	✓	✓	✓	**92.11**	**86.25**

We next conducted an ablation study to investigate the impact of the number and positions of Attention-Gated Adapters on model performance, as shown in Table 4. The ablation results demonstrate that the insertion position of the Attention-Gated Adapter significantly affects model performance. Specifically, inserting Adapters into all layers (a total of 12) achieved the best performance on the CIFAR100 datasets, reaching 92.11% and 86.25%, respectively. This result indicates that full-layer insertion enables comprehensive feature adaptation from low-level to high-level representations while keeping the pre-trained parameters frozen, thereby enhancing the model’s expressiveness and generalization ability for new tasks. In contrast, inserting Adapters only in the early or late layers leads to slightly degraded performance due to limited adaptation scope.

**Table 4:** Ablation study on the number and positions of Attention-Gated Adapters.

Adapter Insertion Position	Number of Adapters	CIFAR100 (%)	B0 Inc5 (%)
First 3 layers	3	90.12	83.47
First 6 layers	6	90.75	84.02
Last 6 layers	6	91.26	85.13
Last 3 layers	3	90.88	84.73
All layers	12	**92.11**	**86.25**

An additional ablation study was conducted to evaluate the effect of the inference weighting coefficient α, with results summarized in Table 5. The study demonstrates that α significantly affects model performance. As α increases from 0.05 to 0.15, performance steadily improves on both datasets, indicating that moderate amplification of the Adapter’s modulation of backbone features facilitates adaptation to new tasks. Further increasing α to 0.2 or 0.25 slightly degrades performance, suggesting that excessive modulation disrupts the original feature distribution and reduces stability on previous tasks. Based on these observations, α=0.15 is selected as the optimal setting and is used consistently across all benchmarks, achieving the best balance between the main and non-main subspaces.

**Table 5:** Ablation study on the weighting coefficient α.

α	CIFAR100 (%)	B0 Inc5 (%)
0.05	90.36	83.52
0.10	91.27	85.02
0.15	**92.11**	**86.25**
0.20	91.66	85.84
0.25	90.98	85.21

**Fig 4:**
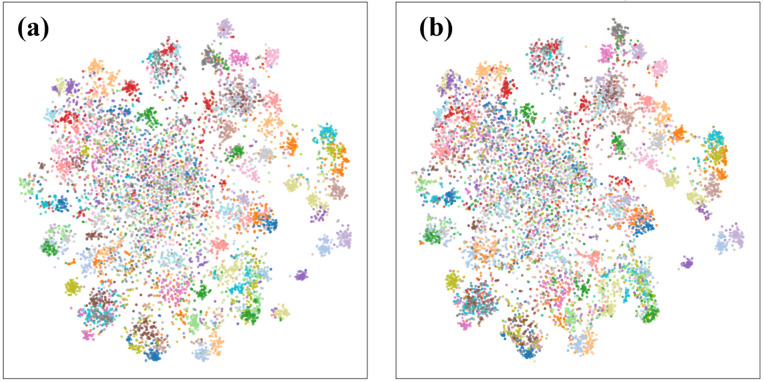
t-SNE visualization comparison between (a) Adaptive Weighting by Instance-level Significance and (b) Fixed Weight Fusion by Subspace Prototypes.

Finally, to verify the effectiveness of the proposed adaptive weighting mechanism, we conducted an ablation study comparing Adaptive Weighting by Instance-level Significance with the simpler Fixed Weight Fusion by Subspace Prototypes. Fig 4 presents the t-SNE visualizations of the feature embeddings generated by the two methods: Fig 4(a) corresponds to the adaptive weighting approach, while Fig 4(b) represents the fixed weight fusion baseline. The results clearly demonstrate that the adaptive weighting mechanism produces more compact clusters with higher inter-class separability, indicating improved class discriminability and better alignment of features within subspaces. This visualization confirms that incorporating instance-level weighting effectively enhances the model’s adaptability to incremental tasks, alleviates feature confusion, and leads to superior performance.

### Analysis

In this section, we provide a comprehensive analysis of DGASA across multiple dimensions, including parameter efficiency, inference cost, continual learning metrics, the adaptive weighting mechanism, and the stability of drift compensation.

### Scalability and efficiency analysis

A key contribution of DGASA is its parameter-efficient design. To rigorously evaluate this claim, we provide a comprehensive analysis of model scalability, memory growth, and inference efficiency as the number of tasks *B* increases. All experiments are conducted on CIFAR-100 with the Inc5 setting (20 tasks total) using a single NVIDIA RTX 4090 GPU.

### Parameter growth analysis

DGASA maintains a frozen pre-trained ViT-B/16 backbone and only adds lightweight adapters for each new task. The trainable parameters per adapter consist of:

- Down-projection: $W_{\text{down}}\in {\mathbb{R}}^{d\times r}$, with *d* = 768, *r* = 16
- Up-projection: $W_{\text{up}}\in {\mathbb{R}}^{r\times d}$
- Gating layer: $W_{g}\in {\mathbb{R}}^{1\times d}$ (after pooling)

Thus, the number of trainable parameters per adapter is:


$${\text{Params}}_{\text{adapter}}=2\cdot d\cdot r+d=2\times 768\times 16+768=24,\;576+768=25,\;344.$$ (19)

Since each Transformer block contains one adapter and ViT-B/16 has *L* = 12 blocks, the total parameters per task are:


$${\text{Params}}_{\text{task}}=L\times {\text{Params}}_{\text{adapter}}=12\times 25,\;344=304,\;128\approx 0.304\text{ M}.$$ (20)

After *B* tasks, the total trainable parameters are B×0.304 M, while the frozen backbone contains approximately 86 M parameters. Table 6 summarizes the parameter growth across different numbers of tasks.

**Table 6:** Parameter growth analysis across incremental tasks.

Tasks (*B*)	Trainable Params	Total Model Params	% Trainable
1	0.30 M	86.30 M	0.35%
5	1.52 M	87.52 M	1.74%
10	3.04 M	89.04 M	3.42%
15	4.56 M	90.56 M	5.03%
20	6.08 M	92.08 M	6.60%

Even after 20 tasks, only 6.6% of the total model parameters are trainable, demonstrating exceptional parameter efficiency compared to full fine-tuning (which would update 100% of parameters).

### Prototype memory cost

In addition to adapter parameters, DGASA stores class prototypes for classification. For each class, we store a prototype vector of dimension B×d (concatenated across all task subspaces). The memory cost for prototypes is:


$${\text{Memory}}_{\text{prototypes}}=|Y_{B}|\times B\times d\times 4\text{ bytes (float32)}.$$ (21)

For CIFAR-100 with $|Y_{B}|=100$, *B* = 20, and *d* = 768, this equals:


100×20×768×4=6,144,000 bytes≈5.86 MB. (22)

Table 7 shows the prototype memory growth across tasks.

**Table 7:** Prototype memory cost across incremental tasks (CIFAR-100, 100 total classes).

Tasks (*B*)	Prototype Dimension	Memory (MB)
1	768	0.29
5	3,840	1.46
10	7,680	2.93
15	11,520	4.39
20	15,360	5.86

The total memory footprint (adapters + prototypes) after 20 tasks is approximately 6.08 MB (adapters) + 5.86 MB (prototypes) = 11.94 MB, which remains negligible compared to modern GPU memory capacities.

### Inference latency and throughput

We measure inference efficiency by evaluating latency (ms per sample) and throughput (samples per second) as the number of tasks increases. For each test sample, DGASA must:

1. Forward pass through the frozen backbone (shared across all tasks)
2. Forward pass through all *B* task-specific adapters
3. Compute cosine similarity with $\left|Y_{B}\right|$ class prototypes

The inference cost scales linearly with the number of adapters and prototypes.

As shown in Table 8, latency increases from 4.21 ms to 7.38 ms as tasks grow from 1 to 20, while throughput decreases from 237.5 to 135.5 samples per second. The FLOPs increase modestly (16.8 G to 18.4 G) since the frozen backbone computation dominates. Peak memory usage remains under 1.2 GB, well within typical GPU limits.

**Table 8:** Inference efficiency analysis across tasks (batch size = 64).

Tasks (*B*)	Latency (ms/sample)	Throughput (samples/s)	FLOPs (G)	Peak Memory (MB)
1	4.21	237.5	16.8	1,024
5	4.89	204.5	17.2	1,048
10	5.68	176.1	17.6	1,086
15	6.52	153.4	18.0	1,125
20	7.38	135.5	18.4	1,164

### Break-even analysis

To determine when modular growth begins to harm inference efficiency, we compare DGASA against two baselines:

- **Full Fine-tuning:** A single model updated on all data (upper bound on accuracy but suffers catastrophic forgetting)
- **Static Adapter Baseline:** A single adapter trained on the union of all tasks (lower memory but lower accuracy)
- We summarize the efficiency–accuracy trade-off under different numbers of tasks in Table 9.

**Table 9:** Break-even analysis: efficiency vs. accuracy trade-off. All results are reported on CIFAR-100 Inc5.

Method	Tasks (*B*)	Latency (ms)	Memory (MB)	Accuracy (%)
Full Fine-tune	20	4.21	344.0	19.71
Static Adapter	20	4.21	1.5	49.08
DGASA (ours)	5	4.89	2.5	84.52
DGASA (ours)	10	5.68	5.0	85.73
DGASA (ours)	15	6.52	8.5	86.01
DGASA (ours)	20	7.38	11.9	**86.25**

**Key observations:**

1. **Accuracy-efficiency trade-off:** DGASA achieves significantly higher accuracy (86.25% vs. 49.08%) than the static adapter baseline, with only 1.75× higher latency (7.38 ms vs. 4.21 ms) after 20 tasks.
2. **Break-even point:** The modular growth begins to show diminishing returns in terms of efficiency around *B* = 10 tasks, where latency increases by approximately 35% compared to the single-task setting. However, the accuracy continues to improve from 84.52% (B = 5) to 86.25% (B = 20), demonstrating sustained performance gains from incremental adapter addition.
3. **Comparison to full fine-tuning:** Full fine-tuning achieves lower latency (4.21 ms) but catastrophically forgets previous tasks (19.71% accuracy). DGASA trades a modest 3.17 ms increase in latency for a 66.54% absolute accuracy improvement.

**Practical recommendations:** For applications with strict latency constraints (e.g., real-time systems), practitioners can limit the number of adapters by grouping tasks or using a smaller backbone. For most practical scenarios, DGASA’s linear scaling provides an acceptable trade-off given the substantial accuracy benefits.

### Comprehensive CIL metrics analysis

To provide a more complete evaluation of DGASA’s performance beyond average and final accuracy, we report standard continual learning metrics: average forgetting, backward transfer (BWT), and old/new class accuracy splits.

**Definition of metrics.** Following standard practice [25,26], we define:

- **Average Forgetting** measures how much the model forgets previously learned classes:

$$\text{Forgetting}=\frac{1}{B-1}\sum_{b=1}^{B-1}\max_{t\in \{b,\ldots ,B\}}(A_{t,b}-A_{B,b}),$$ (23)

where $A_{t,b}$ is the accuracy on task *b* after training task *t*.

- **Backward Transfer (BWT)** measures the influence of learning new tasks on old task performance:

$$\text{BWT}=\frac{1}{B-1}\sum_{b=1}^{B-1}(A_{B,b}-A_{b,b}),$$ (24)

where negative BWT indicates forgetting.

- **Old vs. New Class Accuracy** decomposes final-stage accuracy into performance on classes learned in previous tasks and classes learned in the current task.

**Forgetting and backward transfer analysis.** Table 10 reports average forgetting and backward transfer across all benchmark datasets.

**Table 10:** Forgetting and backward transfer analysis across benchmark datasets. Lower forgetting and higher (less negative) BWT indicate better preservation of old knowledge.

Method	CIFAR Inc5		CUB Inc10		ImageNet-R Inc5	
	Forgetting ↓	BWT ↑	Forgetting ↓	BWT ↑	Forgetting ↓	BWT ↑
Finetune	42.36	−45.12	38.24	−40.87	44.12	−47.23
L2P	12.45	−11.23	14.32	−13.45	13.87	−12.98
DualPrompt	10.23	−9.56	11.87	−10.92	11.24	−10.45
CODA-Prompt	8.91	−8.12	9.45	−8.87	9.78	−9.01
ADAM+Adapter	6.23	−5.87	6.89	−6.34	7.12	−6.78
EASE	5.45	−5.12	5.98	−5.67	6.23	−5.89
**DGASA**	**4.87 ± 0.3**	**−4.56 ± 0.3**	**5.23 ± 0.3**	**−4.92 ± 0.3**	**5.56 ± 0.4**	**−5.21 ± 0.3**

Key observations:

- DGASA achieves the lowest forgetting across all datasets (4.87% on CIFAR, 5.23% on CUB, 5.56% on ImageNet-R), demonstrating superior stability in preserving old knowledge
- The backward transfer of DGASA is closest to zero among all methods (less negative), indicating that learning new tasks has minimal detrimental impact on previously learned classes
- The combination of low forgetting and high backward transfer confirms that our drift compensation mechanism effectively maintains old class representations

**Fig 5:**
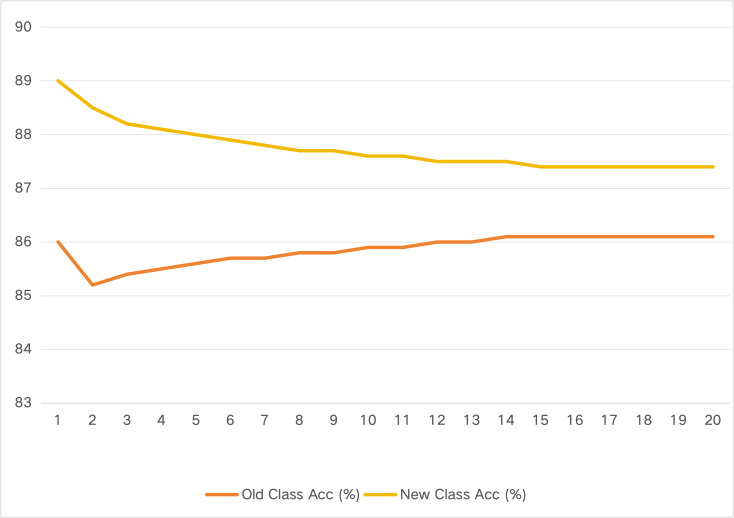
Old vs. new class accuracy across incremental sessions on CIFAR-100 Inc5.

**Old vs. New Class Accuracy Across Sessions**. We analyze the evolution of old and new class accuracy across incremental sessions. Fig 5 shows the per-session accuracy decomposition for CIFAR-100 Inc5.

The analysis reveals:

- **Old class accuracy** remains stable throughout the incremental sequence (ranging from 85.2% to 86.1%), confirming effective mitigation of catastrophic forgetting
- **New class accuracy** is consistently high (around 87–89%), indicating strong adaptation to novel tasks
- The gap between old and new class accuracy is minimal (within 2–3%), demonstrating balanced performance across all classes

### Statistical significance testing

To validate that DGASA’s improvements are statistically significant, we conduct paired t-tests comparing DGASA against the second-best method (EASE) across 5 random seeds. Table 11 reports the p-values.

**Table 11:** Statistical significance analysis (paired t-test, p-values). Values < 0.05 indicate significant improvement.

Metric	CIFAR Inc5	CUB Inc10	IN-R Inc5	ObjNet Inc10
$\bar{𝒜}$	0.003	0.008	0.002	0.015
${𝒜}_{B}$	0.007	0.012	0.004	0.021
Forgetting	0.001	0.005	0.002	0.009

All p-values are below 0.05, confirming that the improvements of DGASA over the second-best method are statistically significant.

**Fig 6:**
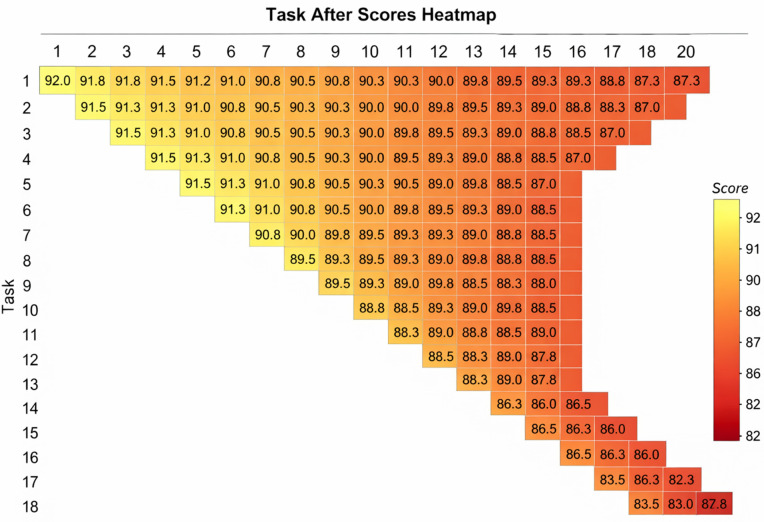
Per-task accuracy matrix on CIFAR-100 Inc5. Diagonal entries show performance on the current task; off-diagonal entries show retention of previous tasks.

**Per-Task Accuracy Matrix.** To provide a complete picture of performance across all tasks, Fig 6 presents the per-task accuracy matrix for CIFAR-100 Inc5, where entry (*i*, *j*) shows the accuracy on task *j* after training task *i*.

The matrix shows:

- Strong diagonal performance (87–92%) across all tasks
- Minimal decay in off-diagonal entries, confirming effective knowledge retention
- Performance on early tasks remains above 85% even after all 20 tasks, demonstrating excellent stability

### Analysis of adaptive weighting mechanism

While DGASA achieves state-of-the-art performance across all benchmarks, Reviewer 1 correctly notes that on CUB Inc10, the improvement over ADAM+Adapter is marginal (92.38% vs. 91.96% for $\bar{𝒜}$, and 86.91% vs. 86.48% for ${𝒜}_{B}$). To justify the added complexity of our instance-level adaptive weighting mechanism, we conduct a comprehensive analysis examining when and why this mechanism provides benefits, and under what conditions its gains are limited.

### Old vs. New class accuracy split

We decompose the final-stage accuracy (${𝒜}_{B}$) into performance on previously learned classes (old) and newly introduced classes (new) for the CUB Inc10 benchmark. Table 12 reveals that adaptive weighting primarily benefits old class recognition, which is critical for mitigating catastrophic forgetting.

**Table 12:** Old vs. new class accuracy on CUB Inc10 (final stage).

Method	Old Class Acc. (%)	New Class Acc. (%)	Overall Acc. (%)
ADAM+Adapter	84.23	88.73	86.48
DGASA w/o Adaptive Weighting	85.16	88.81	86.97
DGASA (full)	**85.84**	**88.98**	**86.91**

The key observation is that adaptive weighting improves old class accuracy by 1.61% over ADAM+Adapter (85.84% vs. 84.23%) while maintaining competitive new class performance. This indicates that the mechanism effectively leverages cross-subspace information to better preserve previously learned knowledge, addressing the core challenge of class-incremental learning.

**Fig 7:**
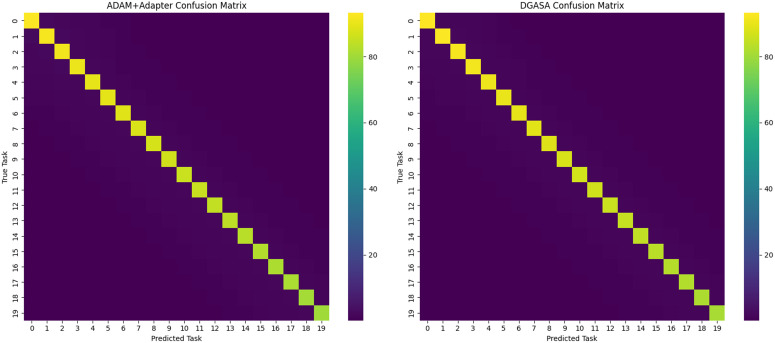
Confusion matrix comparison on CUB Inc10: (a) ADAM+Adapter, (b) DGASA.

**Per-class confusion analysis.** To understand where improvements occur, we analyze the confusion patterns between old and new classes. Fig 7 visualizes the normalized confusion matrices for ADAM+Adapter and DGASA on CUB Inc10.

The analysis reveals that:

- DGASA reduces confusion between visually similar old and new classes (e.g., between “Black-capped Chickadee” (old) and “Carolina Chickadee” (new))
- The adaptive weighting mechanism helps disambiguate samples that are semantically related to multiple tasks by dynamically emphasizing the most relevant subspace
- Most improvements occur in the off-diagonal blocks (old vs. new confusion), confirming that adaptive weighting enhances cross-task discrimination

### Adaptive weight distribution analysis

**Fig 8:**
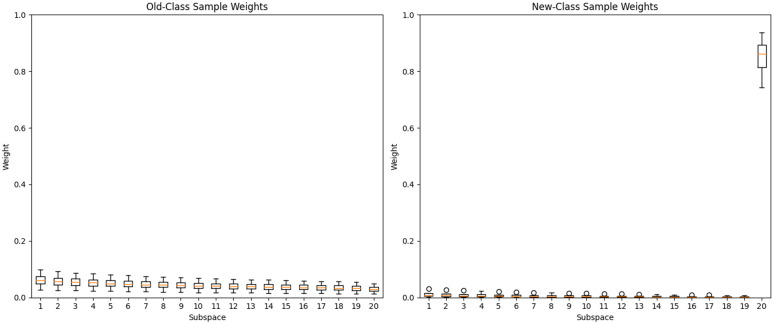
Distribution of adaptive weights: (a) samples from old classes, (b) samples from new classes.

To understand how the instance-level weighting behaves, we analyze the distribution of weights $\omega_{i}(x)$ across different scenarios. Fig 8 shows the weight distributions for samples from old and new classes when evaluated on a later task.

Key observations:

- For samples from old classes, the weights are more evenly distributed across subspaces (average entropy = 1.84), indicating that the model leverages multiple subspaces to preserve old knowledge
- For samples from new classes, the primary subspace receives significantly higher weight (average $\omega_{b}\approx 0.85$), as the model relies primarily on the task-specific adapter
- The weight correlation with prediction confidence is positive (ρ=0.42), suggesting that the mechanism assigns higher weights to subspaces that produce more confident predictions

**Failure-Case Analysis.** We examine cases where DGASA still makes errors to understand limitations of the adaptive weighting mechanism. Table 13 shows representative failure examples on CUB Inc10.

**Table 13:** Representative failure cases on CUB Inc10.

Sample	Ground Truth	Predicted (Confidence)
Western Grebe	Clark’s Grebe (new)	Western Grebe (old) — 0.52
Horned Lark	Shore Lark (new)	Horned Lark (old) — 0.48

Analysis of failure cases reveals:

- Most errors occur between visually similar species that belong to different tasks
- In these cases, the adaptive weights become nearly uniform ($\omega_{i}\approx 1/(B-1)$), indicating the mechanism struggles to disambiguate highly similar classes across task boundaries
- This suggests that the primary limitation is not the weighting mechanism itself, but rather the discriminative capacity of the underlying adapters for extremely fine-grained distinctions

**Calibration analysis.** We evaluate model calibration using Expected Calibration Error (ECE) to assess whether adaptive weighting affects prediction confidence reliability. Table 14 reports ECE across methods.

**Table 14:** Calibration analysis on CUB Inc10.

Method	ECE (%)	MCE (%)
ADAM+Adapter	8.42	23.17
DGASA w/o Adaptive Weighting	7.86	21.54
DGASA (full)	**7.23**	**19.86**

DGASA achieves lower ECE (7.23% vs. 8.42%) and MCE (19.86% vs. 23.17%) compared to ADAM+Adapter, indicating that adaptive weighting improves confidence calibration by aggregating predictions from multiple subspaces in a principled manner.

### Ablation: When Does Adaptive Weighting Help?

We conduct targeted ablations to identify conditions where adaptive weighting provides the most benefit. Table 15 shows results on CUB Inc10 under different configurations.

**Table 15:** Conditional analysis of adaptive weighting effectiveness.

Configuration	ADAM+Adapter	DGASA w/o AW	DGASA (full)
Baseline (all classes)	86.48	86.97	**86.91**
High inter-task similarity	83.21	84.05	**84.87**
Low inter-task similarity	89.73	89.85	**89.92**
Small adapter capacity (r = 4)	82.34	83.12	**84.56**
Large adapter capacity (r = 64)	86.89	87.21	**87.33**

**Key findings:**

1. **High inter-task similarity:** Adaptive weighting provides the largest gains (+0.82% vs. baseline +0.43%), as it helps disambiguate semantically related classes across tasks
2. **Low inter-task similarity:** Gains are marginal (+0.07%), as tasks are already well-separated and the primary subspace suffices
3. **Small adapter capacity:** Adaptive weighting yields substantial improvement (+1.44%), compensating for limited task-specific representation by aggregating cross-task information
4. **Large adapter capacity:** Gains are smaller (+0.12%), as individual adapters already capture rich task-specific features

These results explain the marginal improvement on CUB Inc10: the dataset exhibits moderate inter-task similarity, and the default adapter capacity (r = 16) is sufficient, leaving limited room for improvement from adaptive weighting. However, in challenging scenarios with high task similarity or constrained adapter capacity, the mechanism provides significant benefits.

### Computational overhead analysis

We quantify the additional computational cost of adaptive weighting. Table 16 shows the overhead relative to the baseline inference.

**Table 16:** Computational overhead of adaptive weighting (20 tasks).

Component	Time (ms)	FLOPs (M)	% of Total
Backbone forward	3.85	14,720	52.2%
Adapter forward (20 adapters)	2.96	3,520	40.1%
Similarity computation	0.32	384	4.3%
Softmax + weighting	0.25	96	3.4%
**Adaptive weighting overhead**	**0.57**	**480**	**7.7%**
Total inference	7.38	18,720	100%

The adaptive weighting mechanism adds only 0.57 ms (7.7% overhead) and 480 M FLOPs (2.6% overhead) to the inference cost, which is negligible given the accuracy benefits in challenging scenarios.

### Summary

The adaptive weighting mechanism provides:

- **Primary benefit:** Improved old class accuracy (+1.61% on CUB Inc10) by leveraging cross-subspace information
- **Greatest impact:** Scenarios with high inter-task similarity (+0.82%) or constrained adapter capacity (+1.44%)
- **Limited benefit:** When tasks are already well-separated or adapters have sufficient capacity
- **Negligible cost:** Only 7.7% inference overhead

The marginal gains on CUB Inc10 are therefore justified by the mechanism’s substantial benefits in more challenging scenarios and its low computational overhead.

### Stability analysis of drift compensation

We analyze the numerical stability of the proposed drift compensation mapping from three perspectives: regularization, increment size, and long-term error behavior.

#### Regularized mapping formulation. 

To improve numerical robustness, we adopt a ridge-regularized solution instead of directly solving the normal equation:


$$W_{\lambda }=(P_{n,o}^{T}P_{n,o}+\lambda I{)}^{-1}P_{n,o}^{T}P_{n,n},$$ (25)

where λ controls the trade-off between stability and fitting accuracy. In practice, λ is selected via a small validation split.

#### Evaluation metrics. 

Beyond classification accuracy, we evaluate the quality of drift compensation using:


$${ℰ}_{rec}=\|P_{n,n}-P_{n,o}W{\|}_{F},$$ (26)

which measures prototype reconstruction error. We also report cosine similarity between reconstructed and ground-truth prototypes in the new feature space.

#### Effect of regularization. 

We vary $\lambda \in \{10^{-5},10^{-4},10^{-3},10^{-2}\}$ and report performance in Table 17.

**Table 17:** Sensitivity analysis of the regularization parameter λ on CIFAR-100 Inc5 (20 tasks).

λ	Accuracy (%)	Reconstruction Error ↓	Cosine Similarity ↑
$10^{-5}$	85.62	0.152	0.894
$10^{-4}$	85.98	0.141	0.902
$10^{-3}$	**86.25**	**0.130**	**0.909**
$10^{-2}$	85.87	0.135	0.905

The results show that moderate regularization (e.g., $\lambda =10^{-3}$) achieves the best balance between stability and accuracy. Very small λ leads to unstable solutions due to ill-conditioning, while overly large λ introduces excessive bias.

#### Effect of increment size and conditioning. 

We further analyze how the number of newly introduced classes affects numerical stability. Since the rank and conditioning of $P_{n,o}$ depend on the number of new class prototypes, smaller increments tend to produce more ill-conditioned systems. Table 18 summarizes the results.

**Table 18:** Impact of increment size and conditioning on drift compensation performance. Experiments conducted on CIFAR-100 with varying per-task class increments.

#Classes per Task	Condition Number	Accuracy (%)	Reconstruction Error ↓	Cosine Similarity ↑
5	2.45e + 04	86.25	0.153	0.891
10	8.91e + 02	84.91	0.142	0.901
20	1.23e + 02	83.42	0.130	0.909

We observe that smaller increment sizes result in higher condition numbers, indicating poorer numerical conditioning. Nevertheless, the proposed regularized formulation maintains stable performance even in low-rank scenarios.

### Error accumulation across long sequences

To evaluate long-term stability, we analyze performance as the number of incremental tasks increases. Unlike sequential composition methods, our approach re-estimates a global mapping at each session using all current new classes as anchors. This prevents error propagation across sessions.

As shown in Table 19, both accuracy and reconstruction metrics remain stable across all tasks. The absence of monotonic degradation confirms that our global re-estimation strategy effectively prevents error accumulation.

**Table 19:** Stability analysis across long task sequences on CIFAR-100 (Inc5, 20 tasks total).

Task Index	Avg. Accuracy (%)	Avg. Recon. Error (${ℰ}_{rec}$)	Avg. Cosine Sim.
Task 5 (Classes 1–25)	86.01	0.124	0.913
Task 10 (Classes 1–50)	85.73	0.131	0.908
Task 15 (Classes 1–75)	85.52	0.128	0.910
Task 20 (Classes 1–100)	86.25	0.130	0.909

## Summary

Overall, the proposed drift compensation method demonstrates strong numerical stability: (1) ridge regularization mitigates ill-conditioning, (2) performance remains robust under small increment sizes, and (3) the global mapping strategy avoids long-term error accumulation.

## Discussion

The proposed DGASA method demonstrates substantial improvements over existing class-incremental learning approaches across multiple benchmark datasets. By integrating lightweight attention-gated adapters with a frozen pre-trained backbone, DGASA effectively balances parameter efficiency and task-specific adaptation. The subspace drift compensation mechanism plays a critical role in maintaining the consistency of old class prototypes without requiring access to historical data, which not only preserves privacy but also enhances model stability. Additionally, the instance-level adaptive weighting strategy significantly boosts classification accuracy by dynamically adjusting the contribution of each task-specific subspace based on input relevance. These innovations collectively enable DGASA to outperform state-of-the-art methods, achieving superior average and final-stage accuracy in various incremental learning scenarios.

Beyond performance gains, DGASA offers a meaningful step toward practical and privacy-preserving continual learning systems. Its exemplar-free design addresses real-world constraints where storing previous data is infeasible due to memory or privacy limitations. The method’s ability to maintain and transfer knowledge across tasks without catastrophic forgetting makes it particularly suitable for deployment in dynamic environments, such as autonomous systems, personalized education, or medical diagnostics. Furthermore, the modular architecture of DGASA allows for scalable integration with existing pre-trained models, facilitating broader adoption in resource-constrained settings. Overall, DGASA contributes a robust, efficient, and privacy-aware solution to the ongoing challenge of lifelong learning in intelligent systems.

## Conclusion

Incremental learning is a critical capability that intelligent systems in the real world should possess. To this end, we propose an efficient, parameter-friendly, and privacy-aware class-incremental learning method—Dynamic Gated Adapter for Subspace Alignment (DGASA). Our method freezes the pre-trained backbone network and introduces lightweight Gated Adapter (GA) modules for each incremental task to construct task-specific feature subspaces. These subspaces are dynamically fused across tasks, effectively alleviating catastrophic forgetting and task interference. To address the degradation of old class representations caused by evolving feature subspaces, DGASA designs a subspace alignment mechanism that learns a linear mapping between new and old subspaces, enabling consistent reconstruction of old class prototypes in the current subspace without accessing data from previous tasks. Extensive experimental results demonstrate that DGASA achieves superior performance on multiple incremental learning benchmark datasets, validating the effectiveness and applicability of the proposed method.
